# Development and Evaluation of a Surveillance System for Follow-Up After Colorectal Polypectomy

**DOI:** 10.1001/jamanetworkopen.2023.34822

**Published:** 2023-09-20

**Authors:** Lianlian Wu, Conghui Shi, Jia Li, Zehua Dong, Wei Zhou, Anning Yin, Yanxia Li, Yunchao Deng, Ming Xu, Shan Hu, Jie Pan, Yaowei Ai, Jun Liu, Yijie Zhu, Xiao Tao, Junxiao Wang, Hongliu Du, Xiaoquan Zeng, Honggang Yu

**Affiliations:** 1 Department of Gastroenterology, Renmin Hospital of Wuhan University, Wuhan, Hubei 430060, People’s Republic of China; 2 Hubei Key Laboratory of Digestive System, Renmin Hospital of Wuhan University, Wuhan, Hubei 430060, People’s Republic of China; 3 Hubei Provincial Clinical Research Center for Digestive Disease Minimally Invasive Incision, Renmin Hospital of Wuhan University, Wuhan, Hubei 430060, People’s Republic of China; 4 Engineering Research Center for Artificial Intelligence Endoscopy Interventional Treatment of Hubei Province, Renmin Hospital of Wuhan University, Wuhan, Hubei 430060, People’s Republic of China; 5 School of Computer Sciences of Wuhan University, Wuhan, Hubei 430060, People’s Republic of China; 6 Department of Gastroenterology, Wenzhou Central Hospital, Wenzhou, Zhejiang 325000, People’s Republic of China; 7 Department of Gastroenterology, The People’s Hospital of China Three Gorges University, The First People’s Hospital of Yichang, Yichang, Hubei 443000, P.R. China; 8 Nursing Department of Renmin Hospital of Wuhan University, Wuhan, Hubei 430060, People’s Republic of China

## Abstract

**Importance:**

The adherence of physicians and patients to published colorectal postpolypectomy surveillance guidelines varies greatly, and patient follow-up is critical but time consuming.

**Objectives:**

To evaluate the accuracy of an automatic surveillance (AS) system in identifying patients after polypectomy, assigning surveillance intervals for different risks of patients, and proactively following up with patients on time.

**Design, Setting, and Participants:**

In this diagnostic/prognostic study, endoscopic and pathological reports of 47 544 patients undergoing colonoscopy at 3 hospitals between January 1, 2017, and June 30, 2022, were collected to develop an AS system based on natural language processing. The performance of the AS system was fully evaluated in internal and external tests according to 5 guidelines worldwide and compared with that of physicians. A multireader, multicase (MRMC) trial was conducted to evaluate use of the AS system and physician guideline adherence, and prospective data were collected to evaluate the success rate in contacting patients and the association with reduced human workload. Data analysis was conducted from July to September 2022.

**Exposures:**

Assistance of the AS system.

**Main Outcomes and Measures:**

The accuracy of the system in identifying patients after polypectomy, stratifying patient risk levels, and assigning surveillance intervals in internal (Renmin Hospital of Wuhan University), external 1 (Wenzhou Central Hospital), and external 2 (The First People’s Hospital of Yichang) test sets; the accuracy of physicians and their time burden with and without system assistance; and the rate of successfully informed patients of the system were evaluated.

**Results:**

Test sets for 16 106 patients undergoing colonoscopy (mean [SD] age, 51.90 [13.40] years; 7690 females [47.75%]) were evaluated. In internal, external 1, and external 2 test sets, the system had an overall accuracy of 99.91% (95% CI, 99.83%-99.95%), 99.54% (95% CI, 99.30%-99.70%), and 99.77% (95% CI, 99.41%-99.91%), respectively, for identifying types of patients and achieved an overall accuracy of at least 99.30% (95% CI, 98.67%-99.63%) in the internal test set, 98.89% (95% CI, 98.33%-99.27%) in external test set 1, and 98.56% (95% CI, 95.86%-99.51%) in external test set 2 for stratifying patient risk levels and assigning surveillance intervals according to 5 guidelines. The system was associated with increased mean (SD) accuracy among physicians vs no AS system in 105 patients (98.67% [1.28%] vs 78.10% [18.01%]; *P* = .04) in the MRMC trial. In a prospective trial, the AS system successfully informed 82 of 88 patients (93.18%) and was associated with reduced burden of follow-up time vs no AS system (0 vs 2.86 h).

**Conclusions and Relevance:**

This study found that an AS system was associated with improved adherence to guidelines among physicians and reduced workload among physicians and nurses.

## Key Points

**Question:**

Is an automatic surveillance system associated with improved adherence to guidelines among physicians and reduced workload among physicians and nurses?

**Findings:**

In this diagnostic/prognostic study using endoscopic and pathological reports from 47 544 patients undergoing colonoscopy to develop an automatic surveillance system, the system was associated with increased accuracy among physicians compared with no surveillance system (98.67% vs 78.10%). The automatic surveillance system successfully informed 93.18% of patients and was associated with reduced burden of follow-up time.

**Meaning:**

This study found that an automatic surveillance system was associated with improved adherence to guidelines among physicians and reduced workload of physicians and nurses.

## Teaser

This diagnostic/prognostic study evaluates the accuracy of an automatic surveillance system in stratifying risk levels among patients after colorectal polypectomy.

## Introduction

Colorectal cancer (CRC) is one of the major causes of cancer-related morbidity and mortality worldwide.^1^ Endoscopic screening and removal of colorectal polyps are key strategies to prevent CRC death.^2^ Surveillance colonoscopy is required after polypectomy to detect and resect metachronous and previously misdiagnosed premalignant polyps.^3^ The intensity of surveillance is dependent on endoscopic and histological features of the polyp and also weighed against safety and burden; therefore, several guidelines were issued to classify risk levels of patients after colorectal polypectomy and standardize their follow-up interval, thereby preventing cancer and reducing CRC mortality.^3,4,5,6,7^

However, more than half of patients with a colorectal polyp resected do not receive surveillance colonoscopy within the recommended time.^8^ A study using clinical data found that the overall patient adherence was 48.9% to the 2020 US Multi-Society Task Force polypectomy surveillance guidelines.^9^ A large-scale multicenter study^9^ found that fewer than 25% of patients with adenoma had appropriate surveillance and that patients with delayed surveillance had a much higher rate of advanced neoplasia compared with patients with appropriate surveillance. Asid from patient factors, such as patient economic conditions and insurance-related issues, factors from the physician side are also associated with inappropriate postpolypectomy surveillance. First, physician knowledge and compliance with guidelines vary greatly, associated with heterogeneity in recommendations to patients.^10^ Second, the manual process for tracking patients with different surveillance intervals is energy- and time-consuming, inhibiting active follow-up from physicians and nurses, especially in medical resource–limited areas. Accurate identification of patients with different risk stratification and timely reminders is essential for increasing the surveillance rate of patients after polypectomy and thereby improving early diagnosis.

In recent years, artificial intelligence (AI) has been widely applied in various medical fields.^11^ In the field of AI in gastrointestinal endoscopy, there are numerous studies focusing on computer-aided lesion detection and diagnosis, while few studies pay attention to automatic surveillance for patients.^12^

In this study, we evaluated the accuracy of an automatic surveillance (AS) system in identifying patients after polypectomy from all patients receiving colonoscopy, assigning appropriate surveillance intervals for patients of different risk levels, and proactively following up with patients in time based on patient endoscopic and pathological reports using a deep learning and natural language processing (NLP) method. The performance of AS system was fully evaluated in internal and external test sets according to 5 clinical guidelines (from the US, the UK, China, Europe, and Japan) and compared with that of physicians in clinical practice. Furthermore, a multireader, multicase (MRMC) trial was conducted to evaluate use of the AS system and physician guideline adherence, and prospective data were collected to evaluate success in contacting patients and the association with reduced human workload.

## Methods

This diagnostic/prognostic study follows the Standards for Reporting of Diagnostic Accuracy (STARD) reporting guideline and was approved by the ethics committees of all participating hospitals. For retrospective data sets and follow-up of patients undergoing surveillance colonoscopy, informed consent was exempted by institutional review boards.

### Data Sets

We collected 4 data sets for training and testing of the AS system. A total of 47 544 patients undergoing colonoscopy were included. See the Figure for the study flowchart, including patient inclusion and exclusion criteria.

**Figure.**
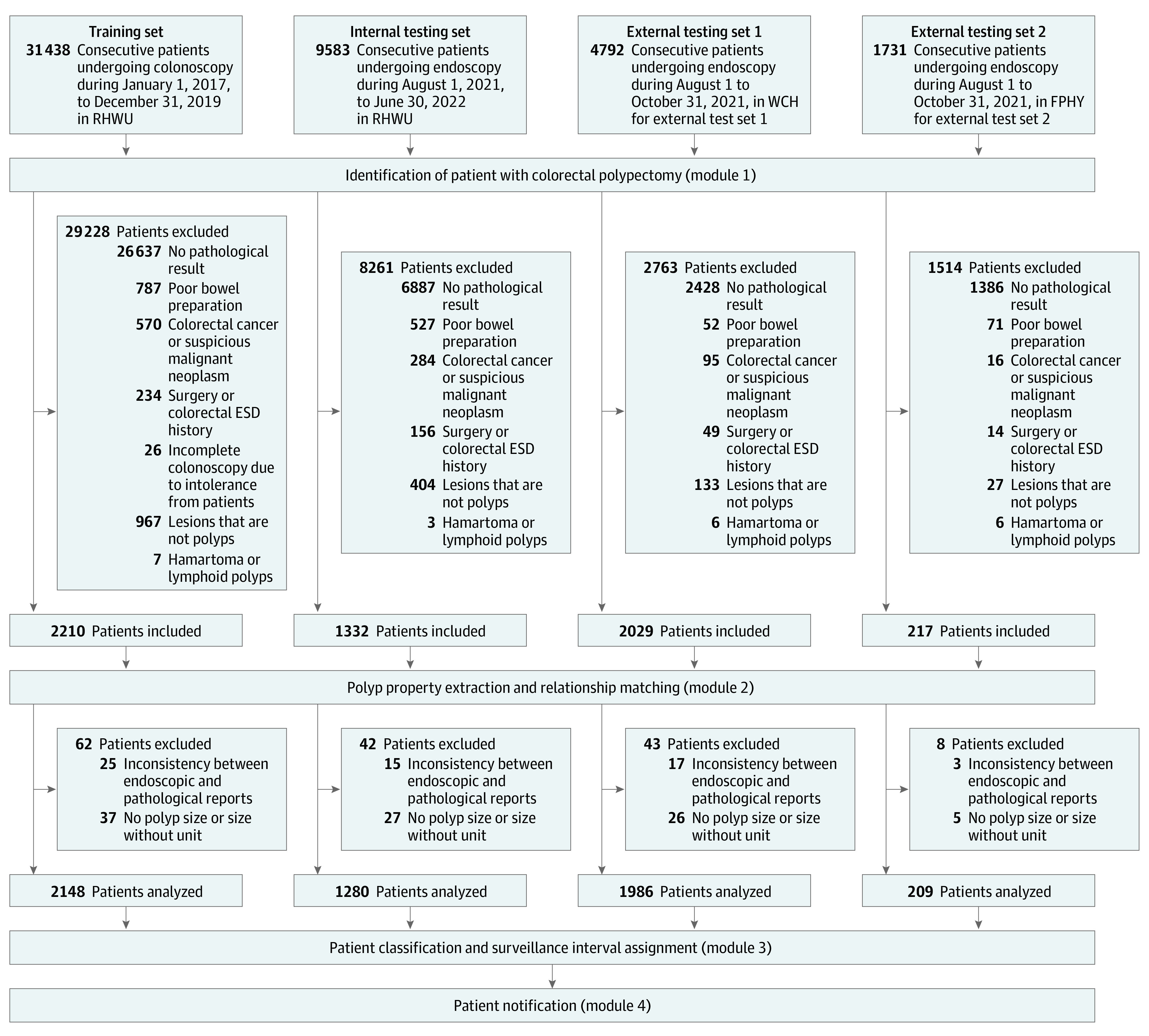
Workflow of Automatic Surveillance System Development ESD indicates endoscopic submucosal dissection; FPHY, The First People’s Hospital of Yichang; RHWU, Renmin Hospital of Wuhan University; WCH, Wenzhou Central Hospital.

In the training set, semistructured endoscopic and pathological text reports (see example in eFigure 1 in Supplement 1) of 31 438 consecutive patients receiving colonoscopy in Renmin Hospital of Wuhan University (RHWU) using the digital endoscopic system Medcare (Medicare Digital Engineering Co, Ltd) from January 1, 2017, to December 31, 2019, were used.

In the internal test set, semistructured endoscopic and pathological text reports of 9583 consecutive patients receiving colonoscopy in RHWU from August 1, 2021, to June 30, 2022, were used. In external test set 1, free-text endoscopic and pathological reports (see example in eFigure 1 in Supplement 1) of 4792 consecutive patients receiving colonoscopy in Wenzhou Central Hospital (WCH) using the digital endoscopic system Kayisoft from August 1 to October 31, 2021, were used.

In external test set 2, semistructured endoscopic and pathological text reports of 1731 consecutive patients receiving colonoscopy in The First People’s Hospital of Yichang (FPHY) using the digital endoscopic system DHC (DHC Software Co, Ltd) from August 1 to October 31, 2021, were used. In RHWU and FPHY, the resect and discard strategy was performed in clinical practice,^13^ which means that diminutive colorectal polyps (≤5 mm) with high-confidence optical diagnosis would be removed and discarded without pathology assessment and other polyps would be sent for pathology. Discarded polyps in enrolled patients were categorized as nonneoplastic based on the endoscopic diagnosis, aligning with the practice observed in clinical setting. In WCH, all removed polyps would be sent for pathology.

### Surveillance Interval Recommendation

To evaluate the adaptability of the AS system, 5 of the latest guidelines from different areas and countries were applied (the Recommendations for Follow-Up After Colonoscopy and Polypectomy: A Consensus Update by the US Multi-Society Task Force on Colorectal Cancer from the US,^3^ Colonoscopy Screening and Surveillance Guidelines from Japan,^4^ Post-Polypectomy Colonoscopy Surveillance: European Society of Gastrointestinal Endoscopy (ESGE) Guideline—Update 2020 from Europe,^5^ British Society of Gastroenterology/Association of Coloproctology of Great Britain and Ireland/Public Health England Post-Polypectomy and Post-Colorectal Cancer Resection Surveillance Guidelines from the UK,^6^ and Expert Consensus on Management Strategies for Precancerous Lesions and Conditions of Colorectal Cancer in China from China^7^). The types of polyps included, patient risk levels, and corresponding surveillance intervals of all guidelines are summarized in eTable 1 in Supplement 1. Based on the 5 guidelines, an expert with more than 10 years of endoscopy experience (H.Y.) reviewed endoscopic and pathological reports of patients and gave a surveillance recommendation serving as the criterion standard.

Patients after polypectomy for whom determining the surveillance interval according to current postcolorectal clinical guidelines was not feasible (eg, those with no pathology or poor bowel preparation) were labeled as “Surveillance interval needs to be manually determined.” This prompted physicians to manually provide surveillance recommendations on the AS system. Subsequently, the AS system could automatically inform patients and endoscopists at crucial times before the given surveillance time, as explained in the Video.

### Development of AS System

The AS system consisted of 4 modules. Module 1 was a regular expression–based model for identifying patients after colorectal polypectomy based on endoscopic and pathological reports. Module 2 consisted mainly of a unified text-to-structure generation framework–based universal information extraction (UIE) model handling endoscopic and pathological reports separately and extracting polyp properties and matching them together. Module 3 was a regular expression–based model that comprehensively analyzed all polyp findings to classify patients into different risk-stratified categories. Module 4 was a text-to-speech^14^ and USB-connected stored program control^15^ exchange technique–based module for automatically sending follow-up messages and making phone calls.

Detailed descriptions of modules are presented in the eMethods in Supplement 1. The workflow and framework for developing the AS system are shown in eFigure 2 and eFigure 3 in Supplement 1, and a case example is presented in eFigure 3 in Supplement 1. The system was developed using the Python programming language version 3.8 (Python Software Foundation).

### Testing and Human-Machine Comparison

We first tested the performance of modules 1 to 3 in the internal test set from RHWU, then assessed AS systems robustness using external test sets 1 and 2 from WCH and FPHY. All endoscopic and pathological reports were evaluated by the expert gastroenterologist (H.Y.) as the criterion standard. Furthermore, to compare the performance between the AS system and physicians, surveillance interval recommendations given by physicians in the discharge advice of enrolled hospitalized patients in the internal test set were extracted and their accuracy was compared with that of AS system. Subgroup analyses were also performed to compare the performance of physicians in different departments with that of the AS system.

### MRMC Trial to Evaluate the AS System and Physician Guideline Adherence

We invited 5 gastroenterologists (2 expert physicians [W.Z. and A.Y.] with >10 years and 3 senior physicians [Y.L., Y.D., and M.X.] with 5-9 years of endoscopic experience) not enrolled in data annotation to participate in the trial. First, they received a training course about the Chinese colorectal postpolypectomy guideline. After 2 weeks, they were required to make surveillance recommendations independently based on patient endoscopic and pathological text reports, which were shuffled and stripped of patient-identifiable information; notably, endoscopic images were also given to physicians for references to fit the clinical environment. After another 2 weeks of a washout period, these experts were asked to make surveillance recommendations for the same patients again (with patient order reshuffled) with the assistance of the AS system. The flowchart, with patient inclusion and exclusion criteria, are shown in eFigure 4 in Supplement 1. The criterion standard came from an expert gastroenterologist (H.Y.) based on Chinese colorectal postpolypectomy guidelines. The primary outcome was the accuracy of physicians in assigning surveillance intervals. Secondary outcomes included the accuracy of the AS system in assigning surveillance intervals and the time cost of physicians making surveillance recommendations.

### Prospective Trial Evaluating Success in Contacting Patients and Association With Reduced Nurse Workload

The AS system first automatically sent follow-up messages and made phone calls to patients based on the criterion standard. Next, 3 nurses were invited to manually make phone calls to inform patients as the positive control (eFigure 5 in Supplement 1), and patients would be asked whether they were informed by the AS system and their preference and acceptance of automatic messages, automatic phone calls, or manual surveillance. Details about questions asked are in the eMethods in Supplement 1. The primary outcome was the success rate of the AS system in informing patients. Secondary outcomes were the success rate for automatically sent messages, answer rate of automatic calls, time cost for nurses of informing patients, and patient acceptance of the system.

### Statistical Analysis

The performance of the AS system was evaluated by accuracy, and the performance of the UIE model was evaluated by sensitivity (the number of correctly predicted polyp descriptions/all polyp descriptions of each attribute) and positive predictive value (the number of correctly predicted polyp descriptions/all predictions of each attribute). The accuracy of physicians and the AS system were compared using the McNemar test. Physician performance with or without the AS system was compared with the Wilcoxon signed-rank test. To measure the agreement among physicians regarding surveillance recommendations, the surveillance interval was first converted to a numeric value based on the median time interval and then evaluated using the intraclass correlation coefficient. The Pearson χ^2^ test was used to evaluate factors correlated with patient preferences. Sample size calculation for the MRMC and prospective trial are described in eMethods in Supplement 1. All reported *P* values are 2-sided, and *P* values < .05 were considered statistically significant. All calculations were performed using SPSS statistical software version 26 (IBM). Data analysis was conducted from July to September 2022.

## Results

The test set included 16 106 patients (7690 females [47.75%]; mean [SD] age, 51.90 [13.40] years). Detailed patient characteristics are shown in eTable 2 in Supplement 1.

### Performance in Identifying Patients Suitable for Clinical Surveillance Guidelines

Patients who were not included in commonly used surveillance guidelines and whose text reports were nonstandard and may have led to misunderstanding were identified by rule-based module 1 with high accuracy (Table 1). In internal, external 1, and external 2 test sets, the overall accuracy of the AS system for distinguishing types of patients was 9574 of 9583 patients (99.91%; 95% CI, 99.83%-99.95%), 4770 of 4792 patients (99.54%; 95% CI, 99.30%-99.70%), and 1727 of 1731 patients (99.77%; 95% CI, 99.41%-99.91%), respectively. Confusion matrixes illustrating module performance on each class are presented in eFigure 6 in Supplement 1.

**Table 1.** Accuracy of the Automatic Surveillance System for Identifying Patient Types

Test set	Patients, No. identified/total No. (%) [95% CI]									
	Overall	Automatic^b^	Manual^a^							
			No pathological result	Poor bowel preparation	Colorectal cancer or suspicious malignant neoplasm	Surgery or colorectal ESD history	Lesions that are not polyps	Hamartoma or lymphoid polyps	Inconsistency between endoscopic and pathological reports	No polyp size or size without unit
Internal (n = 9583)	9574/9583 (99.91) [99.83-99.95]	9577/958 3 (99.94) [99.87-99.97]	9583/958 3 (100) [99.96-100]	9582/958 3 (99.99) [99.94-100]	9580/9583 (99.97 [99.91-99.99]	9581/9583 (99.94) [99.87-99.97]	9580/9583 (99.97 [99.91-99.99]	9583/9583 (100) [99.96-100]	9582/9583 (99.99 [99.94-100]	9581/9583 (99.98) [99.93-99.99]
External 1 (n = 4792)	4770/4792 (99.54) [99.30-99.70]	4778/4792 (99.71) [99.51-99.83]	4792/4792 (100) [99.92-100]	4792/4792 (100) [99.92-100]	4780/4792 (99.75) [99.56-99.86]	4792/4792 (100) [99.92-100]	4790/4792 (99.96) [99.85-99.99]	4791/4792 (99.98) [99.88-100]	4781/4792 (99.77) [99.59-99.87]	4788/4792 (99.92) [99.79-99.97]
External 2 (n = 1731)	1727/1731 (99.77) [99.41-99.91]	1731/1731 (100) [99.78-100]	1727/1731 (99.97) [99.41-99.91]	1731/1731 (100) [99.78-100]	1731/1731 (100) [99.78-100]	1731/1731 (100) [99.78-100]	1729/1731 (99.88) [99.57-99.97]	NA	1729/1731 (99.88) [99.57-99.97]	1731/1731 (100) [99.78-100]

Abbreviations: ESD, endoscopic submucosal dissection; NA, not applicable.

^a^ Patients whose surveillance interval needed to be manually determined or patients who were not included in commonly used surveillance guidelines. These include patients with no pathological result, poor bowel preparation, colorectal cancer or suspicious malignant neoplasm, surgery or colorectal ESD history, lesions that are not polyps, hamartoma or lymphoid polyps, text reports that were nonstandard and would lead to misunderstanding, inconsistency between endoscopic and pathological reports, and no polyp size or size without unit.

^b^ Patients whose surveillance interval could be automatically determined.

### Performance of Module 2 in Extracting Polyp Properties From Text Reports

In endoscopic and pathological text reports of patients analyzed in internal (1280 patients), external 1 (1986 patients), and external 2 (209 patients) test sets, there were 1484 polyp descriptions, 3189 polyp descriptions, and 283 polyp descriptions, respectively, with each description including 4 attributes: polyp location, number, size, and pathology. The UIE model achieved an overall sensitivity of 1445 of 1484 polyp properties (97.37%; 95% CI, 96.43%-98.07%), 2998 of 3189 polyp properties (94.01%; 95% CI, 93.13%-94.78%), and 273 of 283 polyp properties (96.47%; 95% CI, 93.62%-98.07%), respectively, and a positive predictive value of 1445 of 1487 polyp properties (97.18%; 95% CI, 96.21%-97.91%), 2998 of 3189 polyp properties (94.01%; 95% CI, 93.13%-94.78%), and 273 of 284 polyp properties (96.13%; 95% CI, 93.20%-97.83%), respectively, in internal, external 1, and external 2 tests. This model showed good performance on all 4 attributes (eTable 3 in Supplement 1).

### Performance of Module 3 in Stratifying Patient Risk Levels and Assigning Surveillance Intervals

Model 3 achieved an accuracy of at least 99.30% (95% CI, 98.67%-99.63%) on the internal data set, 98.89% (95% CI, 98.33%-99.27%) on external test 1, and 98.56% (95% CI, 95.86%-99.51%) on external test 2 based on all 5 guidelines (Table 2). Confusion matrixes illustrating detailed performance of the module on each classification are presented in eFigures 7 to 9 in Supplement 1.

**Table 2.** Automatic Surveillance System Accuracy in Stratifying Patients by Risk Level Based on Guidelines

Test set	Patients, No. correct/total No. (%) [range]				
	Chinese guideline^7^	US guideline^3^	European guideline^5^	UK guideline^6^	Japanese guideline^4^
Internal (n = 1280)	1274/1280 (99.53) [98.98-99.78]	1275/1280 (99.61) [99.09-99.83]	1277/1280 (99.77) [99.32-99.92]	1271/1280 (99.30) [98.67-99.63]	1278/1280 (99.84) [98.43-99.96]
External 1 (n = 1986)	1964/1986 (98.89) [98.33-99.27]	1970/1986 (99.19) [98.69-99.50]	1976/1986 (99.50) [99.08-99.73]	1974/1986 (99.40) [98.95-99.66]	1971/1986 (99.24) [98.75-99.54]
External 2 (n = 209)	208/209 (99.52 [97.34-99.92]	208/209 (99.52 [97.34-99.92]	206/209 (98.56) [95.86-99.51]	209/209 (100) [98.20-100]	207/209 (99.04) [96.57-99.74]

### Performance of the AS System vs Physicians in Clinical Practice

Among 1280 enrolled patients in the internal test set, there were 1048 patients whose medical records could be traced. The AS system achieved significantly higher accuracy than that of physicians (1047 patients [99.90%; 95% CI, 99.45%-99.99%] vs 165 of 1048 patients [15.74%; 95% CI, 13.66%-18.07%]; *P* < .001). (Table 3) The reasons associated with mistakes of physicians included that they did not give recommendations to patients who needed them (157 patients [14.98%]), gave suggestions for surveillance while not specifying the interval time (207 patients [19.75%]), and shortened time intervals (519 patients [49.52%]). The confusion matrix illustrating detailed performance of the system is presented in eFigure 10 in Supplement 1.

**Table 3.** AS Performance vs Physicians in Assigning Surveillance Intervals

Source	Patients, No. correct/total No. (%)^a^							
	Overall	Risk level 1	Risk level 2	Risk level 3	Risk level 4	Risk level 5	Risk level 6	Risk level 7
AS system	1047/1048 (99.90)	583/583 (100)	13/13 (100)	286/286 (100)	21/21 (100)	129/130 (99.23)	10/10 (100)	5/5 (100)
All physicians	165/1048 (15.74)	7/583 (1.20)	0/13	107/286 (37.41)	10/21 (47.62)	39/130 (30.00)	1/10 (10.00)	1/5 (20.00)
Gastroenterologists	165/928 (17.78)	7/520 (91.35)	0/11	107/249 (42.97)	10/18 (55.56)	39/116 (33.62)	1/10 (10.00)	1/4 (25.00)
Nongastroenterologists	0/120	0/63	0/2	0/37	0/3	0/14	NA	0/1

Abbreviations: AS, automatic surveillance; NA, not available.

^a^ Numerators are the number of patients accurately predicted in each risk level or as a whole. Denominators are the total number of patients in each risk level or as a whole.

Nongastroenterologists had little awareness of giving surveillance recommendations to patients after colorectal polypectomy, with 117 of 120 patients seeing these specialists (97.50%) not given surveillance, while for gastroenterologists, the proportion of patients not given surveillance recommendations was much lower (40 of 928 patients seeing these specialists [431%]). Among patients being given shortened intervals, the mean (SD) shortened time was 1.47 (0.31) years for gastroenterologists and 1.50 (<0.01) years for nongastroenterologists. The detailed performance of physicians in assigning surveillance intervals is presented in eTable 4 in Supplement 1.

### Use of the System and Guideline Adherence Among Physicians

In the MRMC trial, a total of 105 patients (63 males [60.0%]; mean [SD] age, 55.32 [26.53] years) were enrolled (eFigure 4 in Supplement 1). The mean (SD) accuracy and efficiency of 5 gastroenterologists were significantly increased with the assistance of the AS system vs without use of the system (accuracy: 98.67% [1.28%] vs 78.10% [18.01%]; *P* = .04; time cost: 14.03 [5.76] vs 29.0 [20.0] s/patient; *P* = .04), with a greater improvement in on accuracy among nonexperts (risk ratio: 1.37; 95% CI, 1.27-1.46 vs 1.14; 95% CI, 1.08-1.20) (Table 4). The interobserver agreement among gastroenterologists was also improved with vs without use of the system (intraclass correlation coefficient: 0.529; 95% CI, 0.441-0.619 vs 0.819; 95% CI, 0.769-0.863). The detailed performance of 5 physicians is shown in eTable 5 in Supplement 1. Physicians had different personal tendencies when giving interval recommendations, although they were uniformly trained before the test. However, their recommendations become much more homogeneous after using the system.

**Table 4.** Accuracy and Efficiency of Physicians Giving Surveillance Recommendations

Procedure	Correct recommendations, mean (SD) %^a^^,^^b^								Time cost, mean (SD) min^b^
	Overall	Risk							
		Level 1	Level 2	Level 3	Level 4	Level 5	Level 6	Level 7	
Without AS system	78.10 (18.01)	75.83 (36.25)	65.00 (34.69)	93.33 (14.91)	68.57 (31.33)	77.39 (20.25)	100 (0)	50.00 (50.00)	50.75 (35.00)
With AS system	98.67 (1.28)	100 (0)	92.50 (11.18)	100 (0)	98.10 (4.26)	100 (0)	100 (0)	80.00 (27.39)	24.55 (10.08)
*P* value	.04	.07	.11	.32	.07	.07	NA	.08	.04

Abbreviations: AS, automatic surveillance; NA, not applicable.

^a^ Accuracy represents the mean accuracy derived from multiple physician assessments.

^b^ SD is calculated by treating these diverse accuracies as continuous data to reflect the variability among physicians.

### Success of AS System in Contacting Patients and Association With Nurse Workload

All messages were successfully sent by the AS system, and 100 of 105 automatic phone calls (95.24%) were answered, and 67 answered phone calls (67.0%) were meaningful calls (lasting longer than 14 seconds, a period in which the surveillance interval was explained). For manual surveillance, 99 of 105 patients (94.29%) answered phones and 88 patients (83.81%) cooperated with nurses (eFigure 5 in Supplement 1). A total of 2.86 hours was used for nurses to manually inform the 105 patients (mean [SD], 98.4 [46.2] s/patient). With the assistance of the AS system, no manual operation was required for informing patients (0 h).

Among 88 patients analyzed, 82 patients (93.18%) were successfully informed by the AS system and the other 6 patients (6.82%) neither read the automatic messages nor understood the phone voice messages (eFigure 11 in Supplement 1). Among all analyzed patients, 72 patients (81.82%) preferred AI-assisted automatic surveillance, 13 patients (14.77%) preferred manual follow-up, and 3 patients (3.41%) refused to receive any surveillance. Patients aged less than 55 years were more willing to receive automatic surveillance than those aged 55 years or older (odds ratio, 17.73; 95% CI, 2.22-141.43; *P* = .007). Sex and risk level were not associated with patient preferences (eFigure 11 in Supplement 1).

## Discussion

In recent years, AI has seen rapid progress in the application of digestive endoscopy.^16^ NLP, a technology increasingly used to extract data from electronic medical records, has shown good performance as an alternative to manual reporting of endoscopy quality metrics.^17,18,19^ Several previous studies reported the application of NLP in colonoscopy surveillance: Peterson et al^20^ developed a pipeline using text reports from 224 patients and achieved an accuracy of 92% in assigning intervals for surveillance colonoscopy. Karwa et al^21^ constructed a clinical decision–support algorithm and achieved an accuracy of 99% for determining surveillance intervals in 260 patients. It was also reported that a clinical decision support system was associated with improved guideline adherence among physicians for polypectomy surveillance recommendations in a retrospective analysis.^22^ Compared with the previous research, this diagnostic/prognostic study had 3 main novelties. First, the system was implemented in clinical practice and its success was evaluated among health care workers and patients in a prospective trial beyond the technical stage. Second, multicenter data of more than 40 000 patients were used to develop the system, and hospitals with different polyp removal strategies and report structures were specifically selected to evaluate system robustness. Third, 5 clinical guidelines commonly used in assigning surveillance intervals from different countries and areas were implemented to assess system generalizability and adaptability. Results showed that the system achieved good performance in different hospitals in a clinical setting, suggesting that the AS system had considerable transferability. To the best of our knowledge, this is the first study reporting an automatic colonoscopy surveillance system in a multicenter population applied to multiple guidelines from different countries and areas and successfully evaluating its association with reduced burdens of health care workers in a prospective trial.

In clinical practice, physicians give surveillance advice to patients after colorectal polypectomy in discharge records, which is an important reference for patients to arrange surveillance colonoscopy. However, we found that in the clinical environment, the accuracy of giving surveillance interval advice was particularly low (15.74%). Notably, nongastroenterologists had little awareness of giving surveillance advice to patients after polypectomy, which may be associated with the development of potential premalignant polyps; however, except for a few patients with missed recommendations, most gastroenterologists gave advice in shortened intervals, potentially associated with unnecessary overexamination. This is consistent with published research findings^23,24,25^ and is 1 issue that the AS system aims to solve primarily. After training 5 physicians with standard surveillance guidelines, we retested their accuracy and found that it reached 78.10% for surveillance recommendations, suggesting the importance of continual training. Impressively, with the assistance of the AS system, the 5 physicians’ accuracy was further improved to 98.67% and the interphysician consistency was also improved. These findings suggest that the AS system may be associated with improved adherence to guidelines among physicians via reductions in manual errors, illness delays, and unnecessary overtreatment.

In addition to exploring the novel method and evaluating its performance among physicians, we made the last and essential step for the AS system: clinical application for patient follow-up. We designed the system closely according to the clinical use scenario and applied text-to-speech and stored program control exchange techniques to achieve automatic follow-up notification for patients. We explored automatic text messages and automatic calling to inform patients, with 93.18% of patients receiving and understanding notifications and 81.82% of patients preferring AI-assisted automatic surveillance rather than manual calls. Interestingly, older patients were more likely to receive manual calls so they could interact with nurses and ask questions about topics they did not understand, such as why a surveillance colonoscopy should be performed. However, younger patients were more likely to receive an automatic text message, which they thought to be more convenient and efficient. These results may provide evidence for making clinical surveillance strategies and give weight to future related research.

Furthermore, in addition to patients adhering to inclusion criteria of postpolypectomy guidelines, patients for whom determining the surveillance interval according to current guidelines is not feasible (eg, those with poor bowel preparation) may also be semiautomatically monitored by the AS system with the aid of physicians. The AS system temporarily categorized these patients as “Surveillance interval needs to be manually determined” and categorized them in 1 of 8 groups, prompting physicians to manually provide surveillance recommendations on the AS system. Subsequently, the AS system may automatically inform patients and endoscopists at crucial times based on the given surveillance time. This may largely broaden the applicability of the system among patients.

### Limitations

There are also some limitations in our study. First, the system was developed predominantly based on the Chinese language due to data set restrictions. Nevertheless, given that the performance of the system primarily revolved around entity extraction, it may hold theoretical potential for fine-tuning using diverse language data sets, thereby achieving comparable impressive results. Furthermore, exploring the use of contemporary expansive large language models, such as the generative pretrained transformer language model covering multiple languages, may be a prospective avenue for enhancing system generalizability in future endeavors. Second, while the criterion standard aligned with prevailing clinical guidelines, it was established based on the assessment of a single expert gastroenterologist rather than a collective consensus of 2 or more annotators. This situation introduces the possibility of subjectivity influencing outcomes. Therefore, it should be acknowledged that expert evaluation may potentially introduce bias in the accurate identification of patients. Third, despite the improvement the system achieved, it should be noted that the accuracy of physicians in assigning surveillance intervals in clinical practice (15.74%) was exceptionally low. This could be associated with a variety of factors, including instances in which physicians may not have written surveillance intervals in their outpatient or surgical records but may have given oral reminders or sent notification forms. Some surveillance intervals shorter than those of guidelines due to patient conditions may not necessarily be wrong. Consequently, while the accuracy of physicians in assigning surveillance intervals in clinical practice serves as a reasonable metric to unveil the current clinical situation in giving surveillance intervals to patients, it may also exhibit a degree of aggressiveness.

## Conclusions

This diagnostic/prognostic study evaluated an automatic surveillance system aimed at accurately identifying patients after colorectal polypectomy, assigning appropriate surveillance intervals, and proactively following up with patients in time. The system was associated with improved adherence to guidelines among physicians and reduced time spent by physicians and nurses, and it played a role in informing patients. These findings suggest that the system may be associated with improved early diagnosis of premalignant polyps and reductions in unnecessary overexamination and the workload of health care workers.

## Notes

Supplement 1.
eMethods.

eReferences.
**eTable 1.** Surveillance Guidelines for Colorectal Postpolypectomy **eTable 2.** Characteristics of Patients in Test Sets **eTable 3.** Performance of Module 2 in Extracting Polyp Properties **eTable 4.** Detailed Performance of Physicians in Assigning Surveillance Intervals for Inpatients **eTable 5.** Detailed Performance of 5 Physicians With or Without Assistance of Automatic Surveillance System **eFigure 1.** Case Examples of Semistructured and Free-Text Reports **eFigure 2.** Framework of Automatic Surveillance System Development **eFigure 3.** Case Example of Automatic Surveillance System **eFigure 4.** Inclusion and Exclusion Flowchart of Patients in Multireader, Multicase Trial **eFigure 5.** Inclusion and Exclusion Flowchart of Patients in Prospective Follow-Up Trial **eFigure 6.** Confusion Matrixes Illustrating Detailed Performance of Module 1 in Identifying Classes Of Patients **eFigure 7.** Confusion Matrixes Illustrating Detailed Performance of Module 2 in Identifying Risk Levels Among Patients Within Internal Test Set **eFigure 8.** Confusion Matrixes Illustrating Detailed Performance of Module 2 in Identifying Risk Levels Among Patients Within External Test Set 1 **eFigure 9.** Confusion Matrixes Illustrating Detailed Performance of Module 2 in Identifying Risk Levels Among Patients Within External Test Set 2 **eFigure 10.** Confusion Matrixes Illustrating Detailed Performance of Automatic Surveillance System in Assigning Surveillance Intervals for Inpatients **eFigure 11.** Details About Use of Automatic Surveillance System and Patient Preferences by Subgroup

Supplement 2.
Data Sharing Statement
