# Supplementary material for: Development and Evaluation of a Surveillance System for Follow-Up After Colorectal Polypectomy
Source: JAMA Netw Open. 2023 Sep 20;6(9):e2334822. doi: 10.1001/jamanetworkopen.2023.34822 (PMC12278838; doi:10.1001/jamanetworkopen.2023.34822)
Supplement: Supplement 1. — eMethods. eReferences. eTable 1. Surveillance Guidelines for Colorectal Postpolypectomy eTable 2. Characteristics of Patients in Test Sets eTable 3. Performance of Module 2 in Extracting Polyp Properties eTable 4. Detailed Performance of Physicians in Assigning Surveillance Intervals for Inpatients eTable 5. Detailed Performance of 5 Physicians With or Without Assistance of Automatic Surveillance System eFigure 1. Case Examples of Semistructured and Free-Text Reports eFigure 2. Framework of Automatic Surveillance System Development eFigure 3. Case Example of Automatic Surveillance System eFigure 4. Inclusion and Exclusion Flowchart of Patients in Multireader, Multicase Trial eFigure 5. Inclusion and Exclusion Flowchart of Patients in Prospective Follow-Up Trial eFigure 6. Confusion Matrixes Illustrating Detailed Performance of Module 1 in Identifying Classes Of Patients eFigure 7. Confusion Matrixes Illustrating Detailed Performance of Module 2 in Identifying Risk Levels Among Patients Within Internal Test Set eFigure 8. Confusion Matrixes Illustrating Detailed Performance of Module 2 in Identifying Risk Levels Among Patients Within External Test Set 1 eFigure 9. Confusion Matrixes Illustrating Detailed Performance of Module 2 in Identifying Risk Levels Among Patients Within External Test Set 2 eFigure 10. Confusion Matrixes Illustrating Detailed Performance of Automatic Surveillance System in Assigning Surveillance Intervals for Inpatients eFigure 11. Details About Use of Automatic Surveillance System and Patient Preferences by Subgroup [file jamanetwopen-e2334822-s001.pdf]

Supplemental Online Content

Wu L, Shi C, Li J, et al. Development and Evaluation Of A Surveillance System For Follow-Up After Colorectal Polypectomy. *JAMA Netw Open*. 2023;6(9):e2334822. doi:10.1001/jamanetworkopen.2023.34822

eMethods.

eReferences.

eTable 1. Surveillance Guidelines for Colorectal Postpolypectomy

eTable 2. Characteristics of Patients in Test Sets

eTable 3. Performance of Module 2 in Extracting Polyp Properties

eTable 4. Detailed Performance of Physicians in Assigning Surveillance Intervals for Inpatients

eTable 5. Detailed Performance of 5 Physicians With or Without Assistance of Automatic Surveillance System

eFigure 1. Case Examples of Semistructured and Free-Text Reports

eFigure 2. Framework of Automatic Surveillance System Development

eFigure 3. Case Example of Automatic Surveillance System

eFigure 4. Inclusion and Exclusion Flowchart of Patients in Multireader, Multicase Trial

eFigure 5. Inclusion and Exclusion Flowchart of Patients in Prospective Follow-Up Trial

eFigure 6. Confusion Matrixes Illustrating Detailed Performance of Module 1 in Identifying Classes Of Patients

eFigure 7. Confusion Matrixes Illustrating Detailed Performance of Module 2 in Identifying Risk Levels Among Patients Within Internal Test Set

eFigure 8. Confusion Matrixes Illustrating Detailed Performance of Module 2 in Identifying Risk Levels Among Patients Within External Test Set 1

© 2023 Wu L et al. *JAMA Network Open.*

eFigure 9. Confusion Matrixes Illustrating Detailed Performance of Module 2 in Identifying Risk Levels Among Patients Within External Test Set 2

eFigure 10. Confusion Matrixes Illustrating Detailed Performance of Automatic Surveillance System in Assigning Surveillance Intervals for Inpatients

eFigure 11. Details About Use of Automatic Surveillance System and Patient Preferences by Subgroup

This supplemental material has been provided by the authors to give readers additional information about their work.

eMethods

1.1 The details about four modules consisting of automatic surveillance (AS) system

1.1.1 Module 1 for patient identification

Module 1 is a Regular Expression-based model that encoded with the rules to filter out patients who meet the exclusion criteria from the entire patient cohort undergoing colonoscopy based on endoscopic and pathological reports. All patients receiving colonoscopy were included, and the exclusion criteria were as follows: 1) Without pathological result; 2) Poor bowel preparation; 3) Colorectal cancer or suspicious malignancy; 4) Surgery or colorectal ESD history; 5) Patient cannot tolerate colonoscopy and stop early; 6) Lesions of patients are not polyps; 7) Hamartoma or lymphoid polyps. The priority of the exclusion criteria annotation is sorted from 1 to 7; that is, if one patient meets multiple exclusion criteria, he would be labeled as the item with the lowest serial number. An expert gastroenterologist labeled the patients as enrolled or meeting one of the above exclusion criteria. In the process of labeling the training set, the gastroenterologist recorded the key phrases describing each label to form a corpus of each label, upon which the model was developed.

1.1.2 Module 2 for polyp property extraction and polyp grouping

Model 2 contains three models to handle endoscopic and pathological reports separately, and then match them together. Three models are as follows: 1) a unified text-to-structure generation framework based Universal information extraction (UIE) model. The expert gastroenterologist annotated polyp location, number, and size in endoscopic reports using text annotation tool Doccano.[1] We conducted fine-tuning of on UIE model on tokenizing the polyp property within endoscopic reports. The training dataset was divided into an 8:2 ratio for fine-tuning and validation of the UIE model.. The learning rate was set to be 1e-5, with a batch size of 32. The text maximum segmentation length was set at 512 tokens, with training

© 2023 Wu L et al. *JAMA Network Open.*

epochs of 50. The model was trained based on PaddlePaddle 2.3.2 framework. 2) Regular Expression-based polyp pathology extraction model. The expert gastroenterologist annotated the location and corresponding pathological results in pathological reports, and recorded the keywords about locations, forming a corpus of polyp locations in pathological reports. This corpus was subsequently transformed into rules for the model. Once a location was identified, the corresponding pathological result could be determined based on the location's index; 3) Rule-based polyp grouping model. The properties (location, number, size, and pathology) of the same group of polyps were integrated by their index and via matching the locations in endoscopic and pathological reports. If a polyp is a serrated lesion or the size is over 20mm, the keywords "sessile" or "flat" would be further searched between the top and bottom of the index where these polyp properties are located in the endoscopic reports to determine the morphology of serrated lesions and large polyps. Following the processing of module 2, patients whose text reports were nonstandard and may lead to misunderstanding were excluded, which included: 1) Inconsistency between endoscopic and pathological reports; 2) No polyp size or size without unit.

1.1.3 Module 3 for risk level classification and surveillance interval assignment

Module 3, a Regular Expression-based model, comprehensively analyzed all polyp findings extracted from the colonoscopy and pathology reports to classify the patient into different risk-stratified categories, each corresponding to a surveillance interval according to the guidelines.

When stratifying patients to various guidelines, the model was tuned by modifying the rules to align with the distinct risk stratification of each guideline, as outlined in Tables S1A to S1E. For patients with multiple polyps, all the polyps would be assessed adhering to the guidelines' risk levels, starting with the highest and progressing to the lowest. The resulting shortest surveillance interval was subsequently determined and presented as the final recommendation.

© 2023 Wu L et al. *JAMA Network Open.*

1.1.4 Module 4 for automatically sending follow-up messages and making phone calls

Based on patient's examination time and surveillance interval given by module 3, module 4 automatically calculated the period during which patients should undergo surveillance colonoscopy. We pre-set a message template (shown in supplementary materials), and automatically fill each patient's original examination time and recommended surveillance time into the template. Then, the messages were automatically sent to patients using the Short Message Service from Alibaba Cloud (Alibaba, Hangzhou, China). The text messages were then transferred to voice record using TTS (Text to speech) method,[2] and the USB-connected stored program control (SPC) exchange technique[3] was used to achieve automatic phone calls and play the voice record to patients when the call is connected. The time to send messages and make phone calls is set to be one week before the recommended surveillance time. If the patients don't answer after three calls, the call will be stopped.

1.2 Data transferring between hospital's medical system and AS system

The ultimate goal of the system to establish connectivity with the hospital's intranet, seamlessly integrate it into doctors' workstations, and inform patients via off-line communication equipment. However, in the research period, in order to ensure data security and evaluate system performance, we did not connect the AS system to the hospital Intranet, but transferred the data from hospital system into AS system with high efficiency. We firstly listed all the needed anonymized clinical information (including endoscopic and pathological results, examination time and contact information), and then communicated with the manufacturer of the digital endoscopic systems (Medcare [Qingdao, China], Kayisoft system [Zhejiang, China], DHC software system [Beijing, China]) across the three hospitals to give access to the interfaces of those selected data. Consequently, doctors were empowered to simply

© 2023 Wu L et al. *JAMA Network Open.*

confirm patients' examination time and export data of all patients in spreadsheet form by clicking a export button within the endoscopic system, without the need for additional manual operations or daily/weekly manual input. Then the resultant spreadsheet would be imported into the AS system, which automatically analyzes the data and performs surveillance tasks of patients.

1.3 The pre-set message templates

The pre-set message template for automatic message and phone calls is as follows: "Reminders from Endoscopy Center of Renmin Hospital, Wuhan University: You have undergone colorectal polypectomy in our center on {[check: data]}, and a surveillance colonoscopy should be conducted during {[check: data]} and {[check: data]}. Please arrange your time. If you have already taken the surveillance colonoscopy, please ignore this message. Endoscopy Center of Renmin Hospital, Wuhan University cares for your health."

1.4 The questions would be asked to patients in manual calls

1) Have you received and understood the automatically-sent follow-up message?

2) Have you answered and understood the automatic follow-up call?

3) Which surveillance method would you like to receive: AI-assisted or manual; for patients choosing the former, they would be further asked if they prefer automatic messages or call, or both.

If patients don't answer after three calls, the follow-up would be stopped.

1.5 Sample size of MRMC trial

The accuracy of doctors in assigning surveillance intervals was expected to be 70% after training with

© 2023 Wu L et al. *JAMA Network Open.*

guidelines, and increased to 90% with the assistance of AS system, referring to the pilot study. Using the method of the Z test, a sample size of 93 was calculated with a power of 0·90 and a type I rate of 0·025.

1.6 Sample size of prospective trial

The successful rate for informing patients of AS system was expected to be 90% referring to the pilot study. Using the Clopper-Pearson exact method, a sample size of 84 was calculated with a confidence interval width of 0·14 and a confidence level of 95%. A total of 105 patients were required with a dropout rate of 20%.

© 2023 Wu L et al. *JAMA Network Open.*

eReferences

1) Grover M S, Bamdev P, Kumar Y, et al. doccano: Text annotation tool for human. arXiv preprint arXiv 2020; 2006.05236.

2) Zhang H, Mao T, Xu H, Huang H. The NTU-AISG Text-to-speech System for Blizzard Challenge 2020. arXiv preprint arXiv 2020; 2010.11489.

3) Liu, Y S, M Lu. SPC exchange experiment system based on FPGA. Applied Mechanics and Materials,Trans Tech Publications Ltd 2013; 263: 322-328.

1. eTable 1

Table S1A. The surveillance guideline for colorectal post-polypectomy issued by the Chinese Medical Association

Category	Findings	Surveillance interval
1	• Hyperplastic polyps < 10 mm	2-3 years
2	• Sessile serrated polyp < 10 mm without intraepithelial neoplasia	2-3 years
3	• 1-2 tubular adenomas < 10 mm	1-3 years
4	• 3-10 tubular adenomas < 10 mm	1-2 years
5	• Adenoma ≥10 mm<br>• Villous adenoma<br>• Adenoma with high-grade intraepithelial neoplasia.	1-2 years
6	• Sessile serrated polyp ≥10 mm or accompanied by intraepithelial neoplasia;<br>• Traditional serrated polyp;	1-2 years
7	• ≥10 tubular adenomas	1 year

© 2023 Wu L et al. *JAMA Network Open.*

Table S1B. The surveillance guideline for colorectal post-polypectomy issued by US Multi-Society Task Force

Category	Findings	Surveillance interval
**1**	• Hyperplastic polyp < 10mm;<br>• Inflammatory polyp;	10 years
**2**	• 1-2 adenomas <10mm	7-10 years
**3**	• 1-2 serrated polyps < 10mm	5-10 years
**4**	• 3-4 tubular adenomas < 10mm;<br>• 3-4 serrated polyps < 10mm;<br>• Hyperplastic polyp ≥10mm;	3-5 years
**5**	• 5-10 adenomas < 10mm<br>• 5-10 serrated polyps;<br>• Adenoma or serrated polyp ≥10mm<br>• High-grade dysplasia, villous adenoma and tubular villous adenoma;<br>• Traditional serrated adenoma;<br>• Serrated polyp with dysplasia;	3 years
**6**	• > 10 adenomas	1 year
**7**	• Serrated polyp > 2mm	6 months

© 2023 Wu L et al. *JAMA Network Open.*

Table S1C. The surveillance guideline for colorectal post-polypectomy issued by European Society for Gastrointestinal Endoscopy (ESGE)

Category	Findings	Surveillance interval
1	• hyperplastic polyp, < 20mm;<br>• 1-4 adenomas with low grade dysplasia,<br>• < 10 mm, with or without villous dysplasia;<br>• serrated polyp without dysplasia, < 10 mm;	Return to routine colonoscopy screening
2	• Adenoma ≥10 mm, or with high grade dysplasia;<br>• ≥5 adenomas;	3 years
3	• Traditional serrated polyp;<br>• Serrated polyp ≥10 mm;<br>• Serrated polyp with dysplasia;	3 years
4	• Polyp ≥20mm	3-6 months
5	• ≥10 adenomas	Genetic counseling

© 2023 Wu L et al. *JAMA Network Open.*

Table S1D. The surveillance guideline for colorectal post-polypectomy issued by British Society of Gastroenterology (BSG)

Category	Findings	Surveillance interval
**1**	• No high-risk finding*	Return to routine colonoscopy screening
**2**	• ≥2 premalignant polyps#, including at least 1 advanced polyp*	3 years
**3**	• ≥5 precancerous polyps<br>• Non-pedunculated colorectal polyp <20mm	3 years
**4**	• Non-pedunculated colorectal polyp ≥20mm	2-6 months

* High-risk finding: ≥2 premalignant polyps including ≥1 advanced colorectal polyp; or ≥5 premalignant polyps

Premalignant polyps: Adenomas or serrated polyps

* Advanced polyp: Serrated polyp ≥10mm , or serrated polyp with dysplasia (i.e. serrated adenoma), or adenoma ≥10mm, or adenoma with high-grade dysplasia

© 2023 Wu L et al. *JAMA Network Open.*

Table S1E. The surveillance guideline for colorectal post-polypectomy issued by the Japanese Gastroenterological Endoscopy Society (JGES)

Category	Findings	Surveillance interval
**1**	• There is no neoplasia	Go back to the annual FIT screening
**2**	• ≤ 2 adenomas	3-5 years
**3**	• Sessile serrated lesion	3-5 years
**4**	• 3-9 adenomas	3-5 years
**5**	• ≥10 adenomas or with advanced neoplasia *	1-3 years

* Advanced neoplasia: Adenoma ≥10 mm, or with high grade dysplasia; or with villous dysplasia.

© 2023 Wu L et al. *JAMA Network Open.*

2. eTable 2

eTable 2. The characteristics of patients in test sets

Characteristics	Overall (n=16106)	Internal test set (n=9583)	External test set 1 (n=4792)	External test set 2 (n=1731)
**Age, years (SD)**	51.90(13.40)	51.94(13.68)	53.63(12.68)	51.05(12.95)
**Sex, n (%)**				
**Female**	7690(47.75)	4506(47.02)	2372(49.50)	812(46.91)
**Male**	8416(52.25)	5077(52.98)	2420(50.50)	919(53.09)
**Recruitment, n (%)**				
**Inpatient**	6013(37.33)	4376(45.66)	997(20.81)	640(36.97)
**Outpatient**	10093(62.67)	5207(54.34)	3795(79.19)	1091(63.03)
**Category**				
**Risk level 1**	1512	726	678	108
**Risk level 2**	21	18	1	2
**Risk level 3**	1274	336	855	83
**Risk level 4**	187	29	149	9
**Risk level 5**	396	136	254	6
**Risk level 6**	40	11	28	1
**Risk level 7**	10	5	5	0

© 2023 Wu L et al. *JAMA Network Open.*

3. **eTable 3**

eTable 3. The performance of module 2 on extracting polyp properties, % (n/N, 95%CI)

	Overall % (n/N）		Separate							
			Site		Number		Size		Pathology	
	Sensitivity	PPV	Sensitivity	PPV	Sensitivity	PPV	Sensitivity	PPV	Sensitivity	PPV
**Internal test set (n=1484)**	97.37% (1445/1484, 96.43-98.07%)	97.18% (1445/1487, 96.21-97.91%)	99.73% (1480/1484, 99.31-99.89%)	99.53% (1480/1487, 99.03-99.77%)	98.18% (1457/1484, 97.37-98.75%)	98.05% (1458/1487, 97.21-98.64%)	98.68% (1418/1437, 97.95-99.15%)	99.58% (1418/1424, 99.09-99.81%)	99.87% (1482/1484, 99.52-99.97%)	99.66% (1482/1487, 99.21-99.85%)
**External test set 1 (n=3189)**	94.01% （2998/3189, 93.13-94.78%）	94.01% （2998/3189, 93.13-94.78%）	99.97% (3188/3189, 99.82-99.99%)	99.97% (3188/3189, 99.82-99.99%)	95.36% (3039/3187, 94.57-96.04%)	95.30% (3039/3189, 94.51-95.98%)	97.16% (2977/3064, 96.51-97.69%)	99.20% (2976/3000, 98.81-99.46%)	99.72% (3180/3189, 99.47-99.85%)	99.75% (3180/3188, 99.51-99.87%)
**External test set 2 (n=283)**	96.47% (273/283, 93.62-98.07%)	96.13% (273/284, 93.20-97.83%)	100% (283/283, 98.66-100%)	99.65% (283/284, 98.04-99.94%)	97.52% (275/282, 94.97-98.79%)	97.18% (276/284, 94.54-98.56%)	98.05% (251/256, 95.51-99.16%)	100% (249/249, 98.48-100%)	99.65% (282/283, 98.03-99.94%)	99.30% (282/284, 97.48-99.81%)

© 2023 Wu L et al. *JAMA Network Open.*

4. eTable 4

eTable 4. The detailed performance of doctors on assigning surveillance interval for inpatients

		**All doctors**	**Gastroenterologists**	**Non-gastroenterologists**
**Overall**	Correct % (n/N)	15.74% (165/1048)	17.78% (165/928)	0 (0/120)
	Interval not given % (n/N)	14.98% (157/1048)	4.31% (40/928)	97.50% (117/120)
	Interval not specified % (n/N)	19.75% (207/1048)	22.20% (206/928)	0.83% (1/120)
	Interval shortened % (n/N), average shortened time ± SD	49.52% (519/1048), 1.47 ± 0.31 y	55.71% (517/928), 1.47 ± 0.31 y	1.67% (2/120), 1.5 ± 0 y
**Risk level 1**	Correct % (n/N)	1.20% (7/583)	1.35% (7/520)	0 (0/63)
	Interval not given % (n/N)	13.55% (79/583)	3.46% (18/520)	96.82% (61/63)
	Interval not specified % (n/N)	19.73% (115/583)	22.11% (115/520)	——
	Interval shortened % (n/N), average shortened time ± SD	65.52% (382/583), 1.66 ± 0.26 y	73.08% (380/520), 1.57 ± 0.27 y	3.17% (2/63), 1.5 ± 0 y
**Risk level 2**	Correct % (n/N)	0 (0/13)	0 (0/11)	0 (0/2)
	Interval not given % (n/N)	15.38% (2/13)	90.91% (10/11)	0 (0/2)
	Interval not specified % (n/N)	7.69% (1/13)	9.09% (1/11)	——
	Interval shortened % (n/N), average shortened time ± SD	76.92% (10/13), 1.58 ± 0.39 y	——	——
**Risk level 3**	Correct % (n/N)	37.41% (107/286)	42.97% (107/249)	0 (0/37)
	Interval not given % (n/N)	17.83% (51/286)	6.02% (15/249)	0 (0/37)
	Interval not specified % (n/N)	21.68% (62/286)	24.50% (61/249)	——
	Interval shortened % (n/N), average shortened time ± SD	23.08% (66/286), 1.30 ± 0.37 y	26.51% (66/249), 1.30 ± 0.37 y	——
**Risk level 4**	Correct % (n/N)	47.62% (10/21)	55.56% (10/18)	0 (0/3)
	Interval not given % (n/N)	23.81% (5/21)	11.11% (2/18)	0 (0/3)

© 2023 Wu L et al. *JAMA Network Open.*

	Interval not specified % (n/N)	9.52% (2/21)	11.11% (2/18)	——
	Interval shortened % (n/N), average shortened time ± SD	19.05% (4/21), 1.0 ± 0.20 y	22.22% (4/18), 1.0 ± 0.20 y	——
**Risk level 5**	Correct % (n/N)	30.00% (39/130)	33.62% (39/116)	0 (0/14)
	Interval not given % (n/N)	13.85% (18/130)	3.44% (4/116)	0 (0/14)
	Interval not specified % (n/N)	16.15% (21/130)	18.10% (21/116)	——
	Interval shortened % (n/N), average shortened time ± SD	40.00% (52/130), 1.07 ± 0.19 y	44.83% (52/116), 1.07 ± 0.19 y	——
**Risk level 6**	Correct % (n/N)	10.00% (1/10)	10.00% (1/10)	——
	Interval not given % (n/N)	——	——	——
	Interval not specified % (n/N)	50.00% (5/10)	50.00% (5/10)	——
	Interval shortened % (n/N), average shortened time ± SD	40.00% (4/10), 1.03 ± 0.21 y	40.00% (4/10), 1.03 ± 0.21 y	——
**Risk level 7**	Correct % (n/N)	20.00% (1/5)	25.00% (1/4)	0 (0/1)
	Interval not given % (n/N)	40.00% (2/5)	25.00% (1/4)	0 (0/1)
	Interval not specified % (n/N)	20.00% (1/5)	25.00% (1/4)	——
	Interval shortened % (n/N), average shortened time ± SD	20.00% (1/5), 0.5 y	25.00% (1/4), 0.5 y	——

© 2023 Wu L et al. *JAMA Network Open.*

5. eTable 5

eTable 5. The detailed performance of the five doctors with or without the assistance of automatic surveillance (AS) system

	Experts		Non-experts		
	**Doctor A**	**Doctor B**	**Doctor C**	**Doctor D**	**Doctor E**
**Without AS system**					
Correct n (%)	84 (80.00%)	94.29% (99/105)	54 (51.43%)	94.29% (99/105)	74 (70.48%)
Interval shortened n (%), average shortened time ± SD	9 (8.57%), 1.06 ± 0.17 y	1 (0.95%), 0.5 y	——	4 (3.81%), 1.5 ± 0 y	31 (29.53%), 1.24 ± 0.25 y
Interval delayed n (%), average delayed time ± SD	12 (11.43%), 1.04 ± 0.14 y	5 (4.76%), 1.17 ± 0.29 y	51 (48.57%), 1.93 ± 0.49 y	2 (1.90%), 1.5 ± 0 y	——
**With AS system**					
Correct n (%)	100% (105/105)	98.10% (103/105)	97.14% (102/105)	100% (105/105)	98.10% (103/105)
Interval shortened n (%), average shortened time ± SD	——	1 (0.95%), 1 y	——	——	2 (1.90%), 1.5 ± 0 y
Interval delayed n (%), average delayed time ± SD	——	1 (0.95%), 0.5 y	3 (2.86%), 1.67 ± 0.29 y	——	——

© 2023 Wu L et al. *JAMA Network Open.*

6. eFigure 1

A: Semi-structured text reports

Endoscopic reports

Colonoscopy examination was performed under ECG monitoring. 5 minutes were taken to insert the colonoscope into **ileocecal junction**. No abnormality was found in the ileocecal junction. The ileocecal valve was lip-shaped; the appendix opening was crescent-shaped.
Ascending colon and hepatic flexure: The bowel lumens were triangular, mucosa was smooth, and the vascular network was clear;
Transverse colon: The bowel lumens were triangular, the mucosa was smooth, and the vascular network was clear. A flat bulge of about 0.3*0.4cm in size was seen near the splenic flexure (about 50cm from the anus). A disposable biopsy forceps was used to take it and the sample was send for pathological examination. After treatment, no bleeding was observed for a few minutes.
Descending colon and splenic flexure: The bowel lumens were triangular, mucosa was smooth, and the vascular network was clear;
Sigmoid colon: The bowel lumens were round, the mucosa was smooth, and the vascular network was clear;
Rectum: The rectal mucosa was smooth and the vascular network was clear. Hemorrhoids were seen in the anus.
Endoscopic diagnosis: Colon polyp removal with forceps.

Pathological reports

Transverse colon: Villous adenoma with low-grade intraepithelial neoplasia.

B: Free-text reports

Endoscopic reports

The colonoscope was inserted to the end of the ileum. The insertion process took 6 minutes; the lip-shaped ileocecal valve and the semilunar appendix opening were seen. There was no abnormity in the mucosa of terminal ileum, ileocecum, ascending colon, transverse colon, descending colon. A flat polyp about 0.4cm with smooth surface was seen in the sigmoid colon at about 35 cm to the anus, which was removed by forceps. A flat polyp of about 0.6cm with smooth surface was seen in the rectum at about 15 cm to the anus, and it was strangled with a snare. The wound was slightly bleeding, and was clamped with titanium clips.

Endoscopic diagnosis: Colorectal polyps.

Pathological reports

Hyperplastic polyps in sigmoid colon and rectum

eFigure 1. The case examples of semi-structured and free-text reports.

© 2023 Wu L et al. *JAMA Network Open.*

7. **eFigure2**

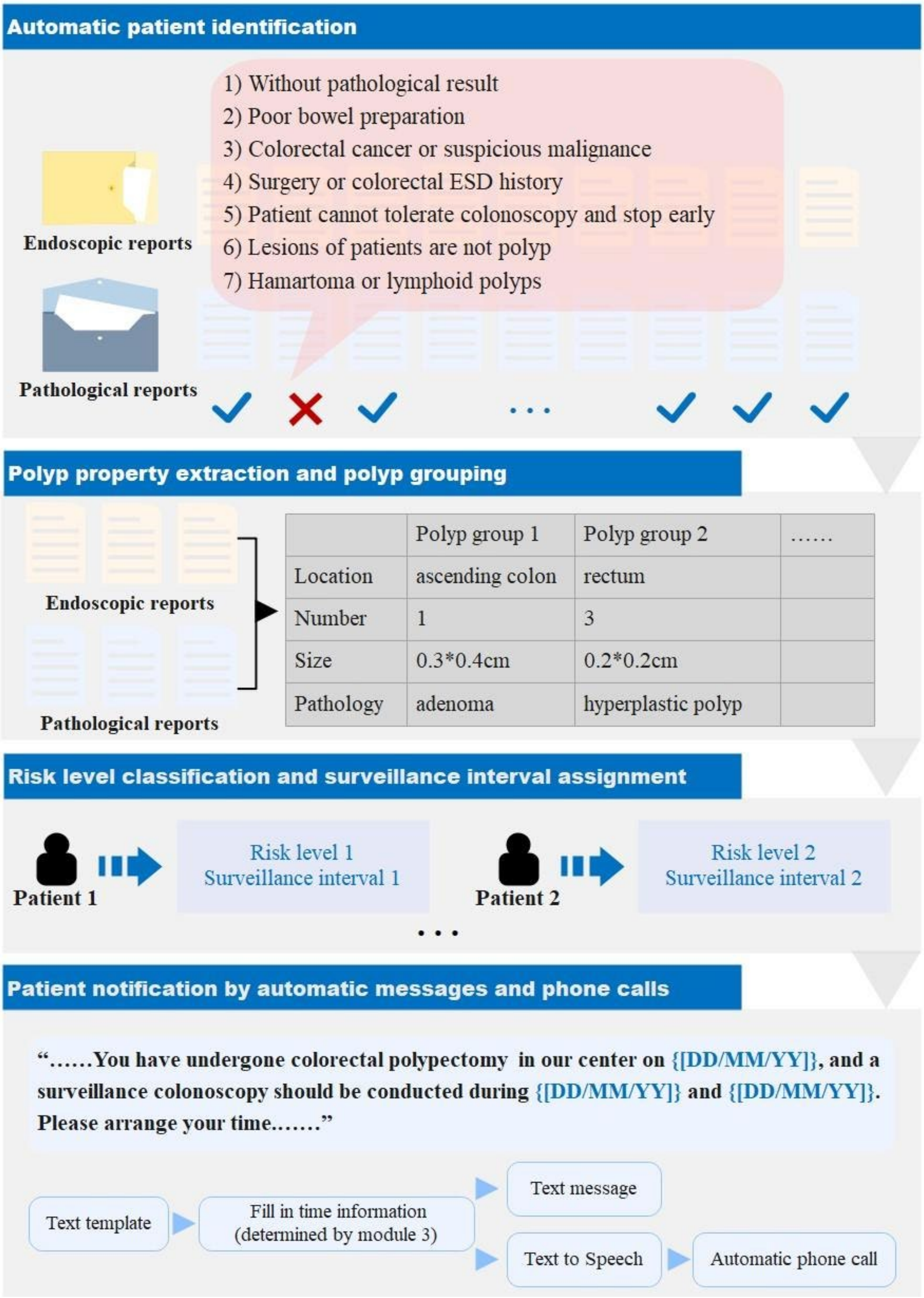

eFigure 2. The framework of developing automatic surveillance (AS) system.

8. eFigure 3

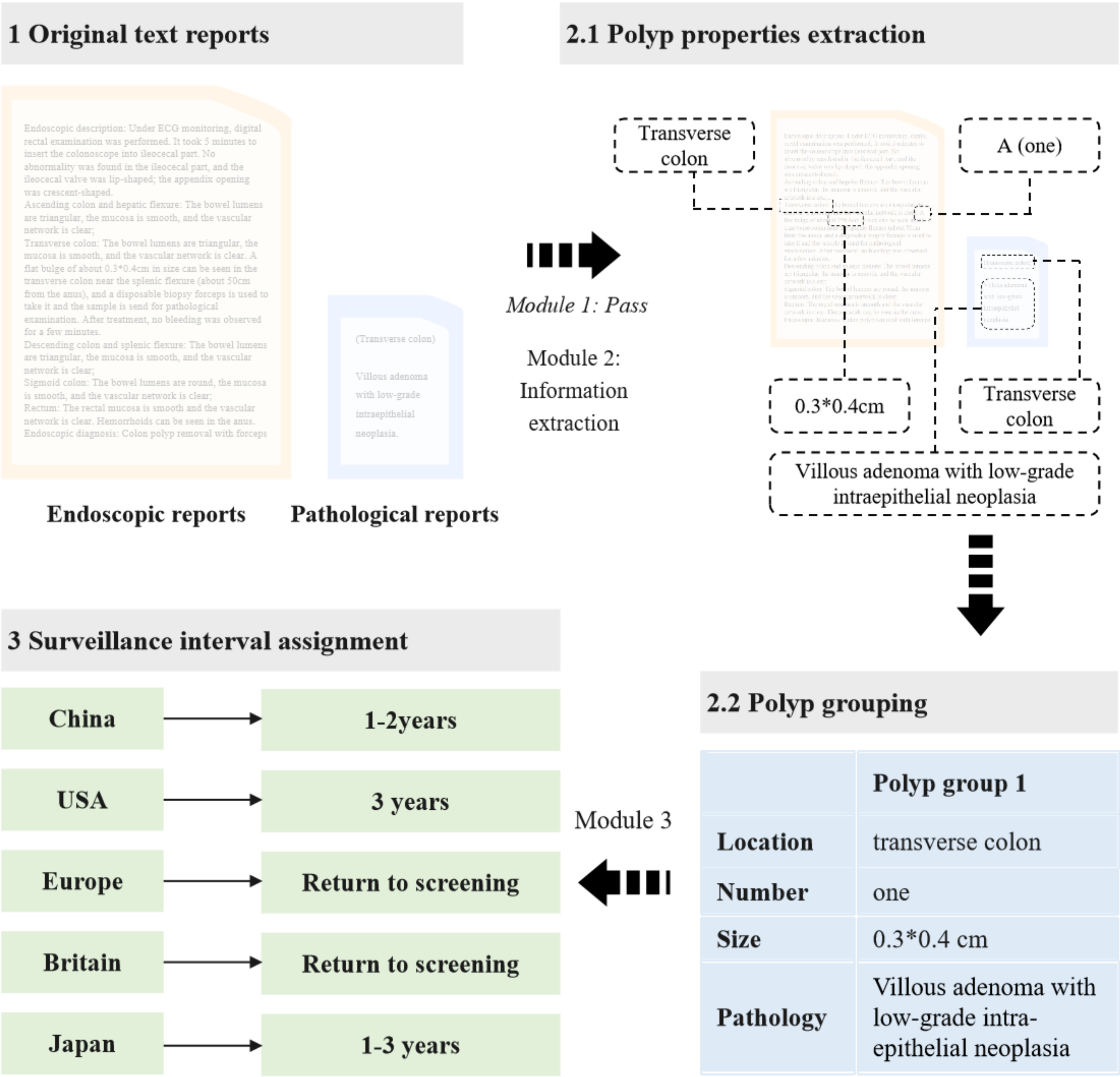

eFigure 3. A case example of automatic surveillance (AS) system. ESD: endoscopic submucosal dissection.

© 2023 Wu L et al. *JAMA Network Open.*

9. eFigure 4

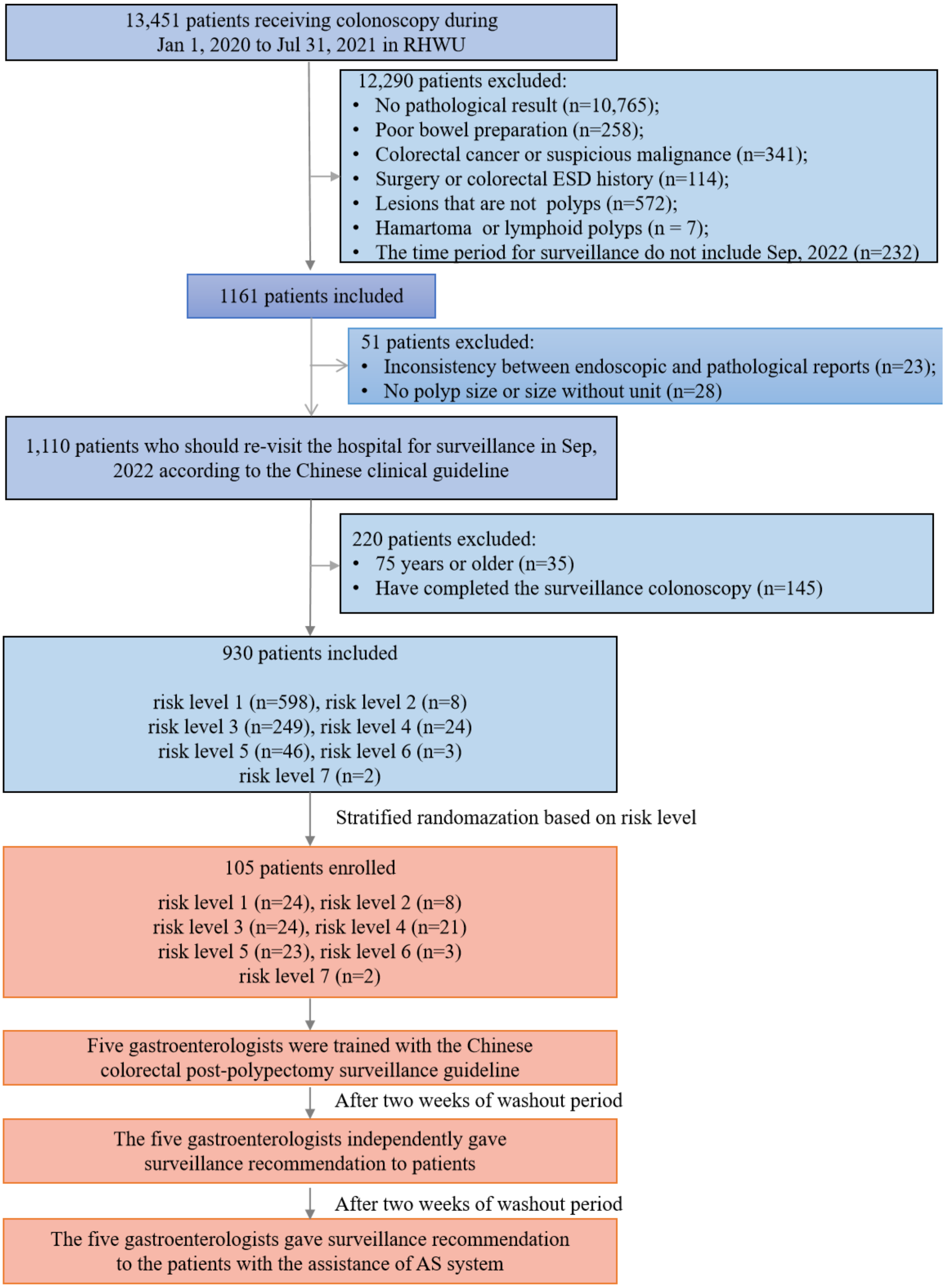

eFigure 4. The inclusion and exclusion flow chart of patients in the multi-reader-multi-case trial.

© 2023 Wu L et al. *JAMA Network Open.*

10. eFigure 5

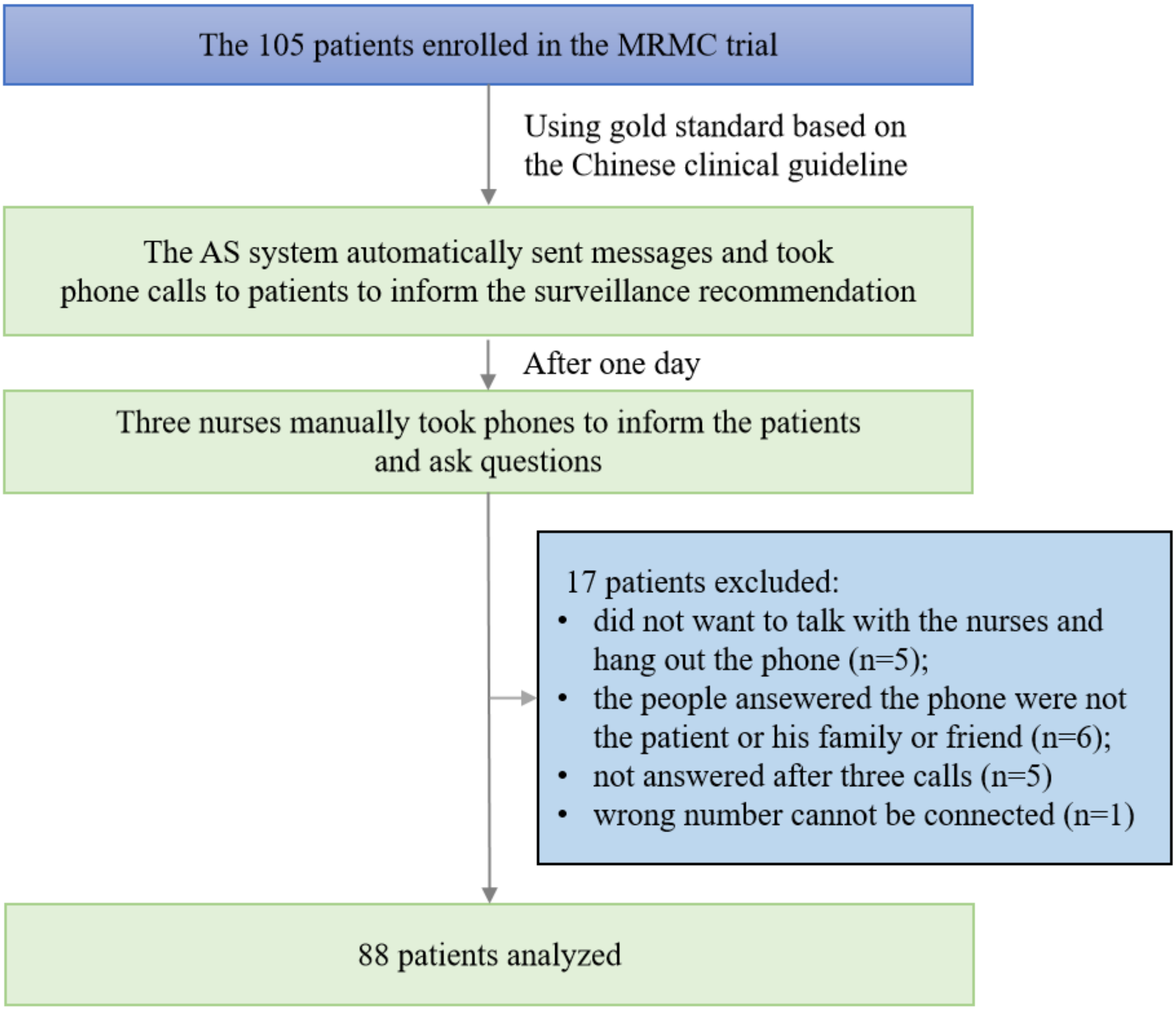

eFigure 5. The inclusion and exclusion flow chart of patients in the prospective follow-up trial.

© 2023 Wu L et al. *JAMA Network Open.*

11. eFigure6

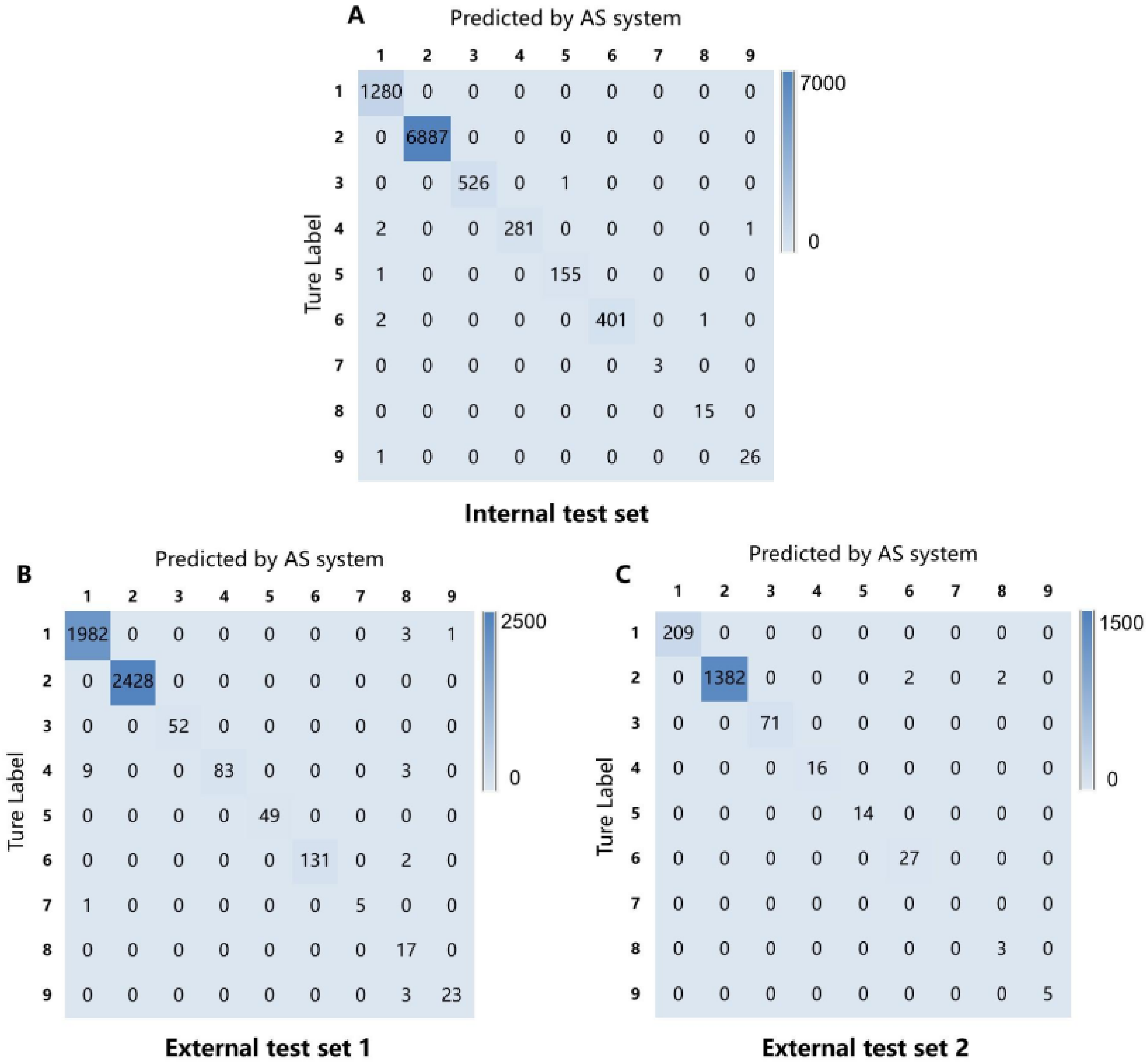

eFigure 6. The confusion matrixes illustrating detailed performance of module 1 on identifying different classes of patients. 1: Surveillance interval can be automatically determined; 2: No pathological result; 3: Poor bowel preparation; 4: Colorectal cancer or suspicious malignance; 5: Surgery or colorectal ESD history; 6: Lesions that are not polyps; 7: Hamartoma or lymphoid polyps; 8: Inconsistency between endoscopic/pathological reports9: No polyp size or size without unit. A. The performance of module 1 in the internal set; B. The performance of module 1 in external set 1; C. The performance of module 1 in external set 2.

© 2023 Wu L et al. *JAMA Network Open.*

12. eFigure 7

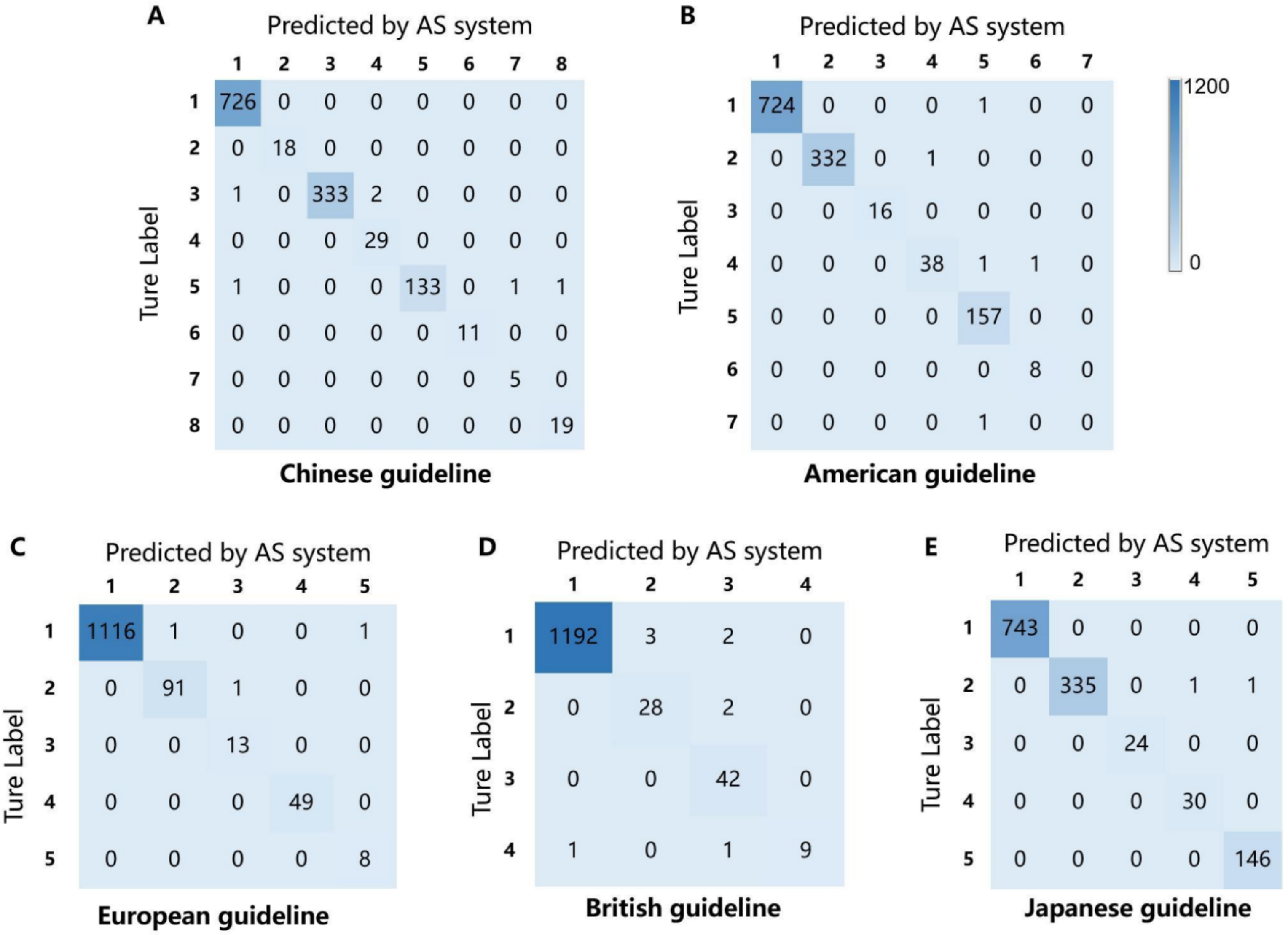

eFigure 7. The confusion matrixes illustrating detailed performance of module 2 on identifying different risk levels among patients within the internal test set, in accordance with five distinct clinical guidelines. The labels of categories 1-8 are explained in Table S1A – S1E.

© 2023 Wu L et al. *JAMA Network Open.*

13. eFigure 8

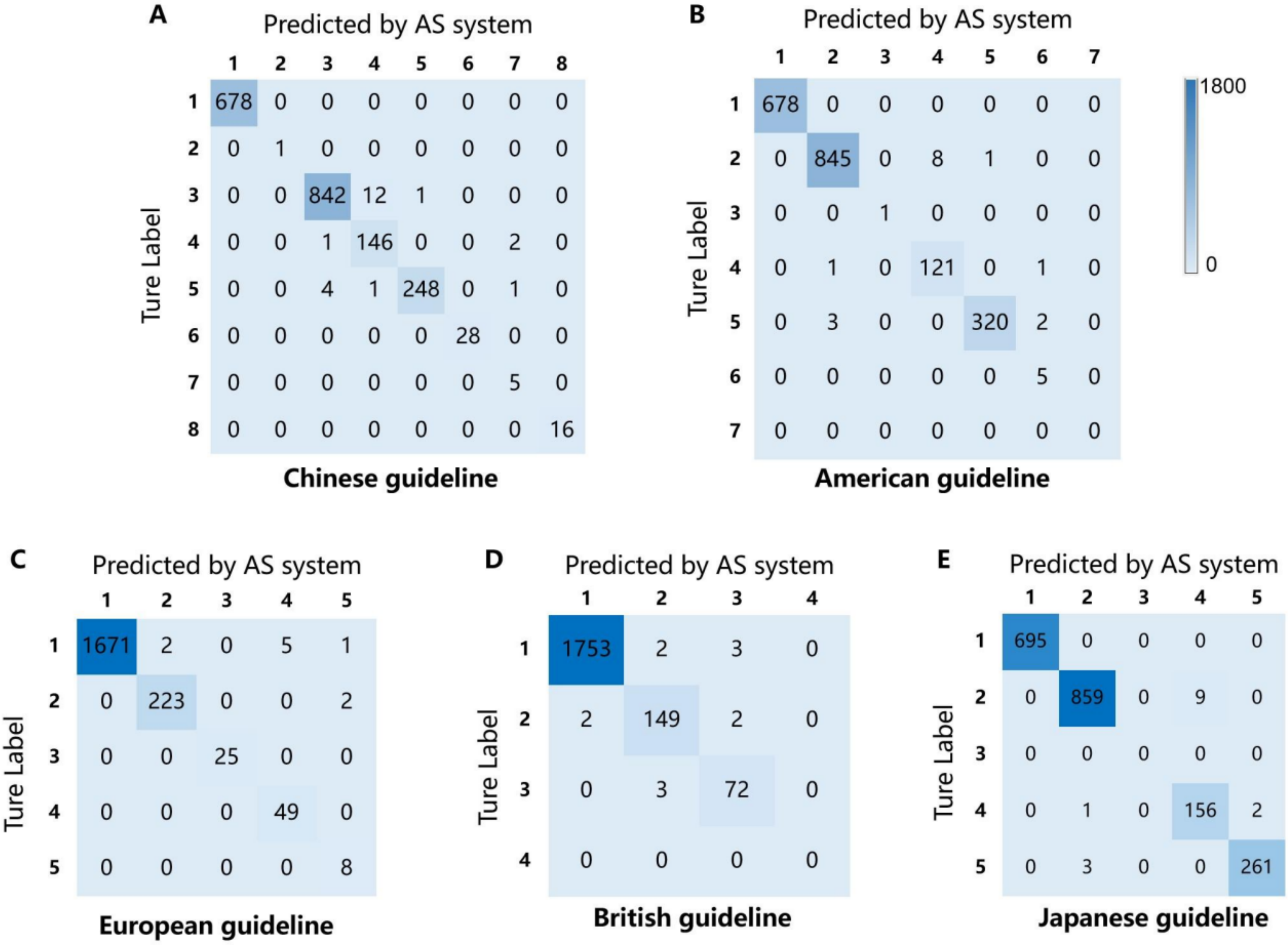

eFigure 8. The confusion matrixes illustrating detailed performance of module 2 on identifying different risk levels among patients within the external test set 1, in accordance with five distinct clinical guidelines. The labels of categories 1-8 are explained in Table S1A – S1E.

© 2023 Wu L et al. *JAMA Network Open.*

14. eFigure 9

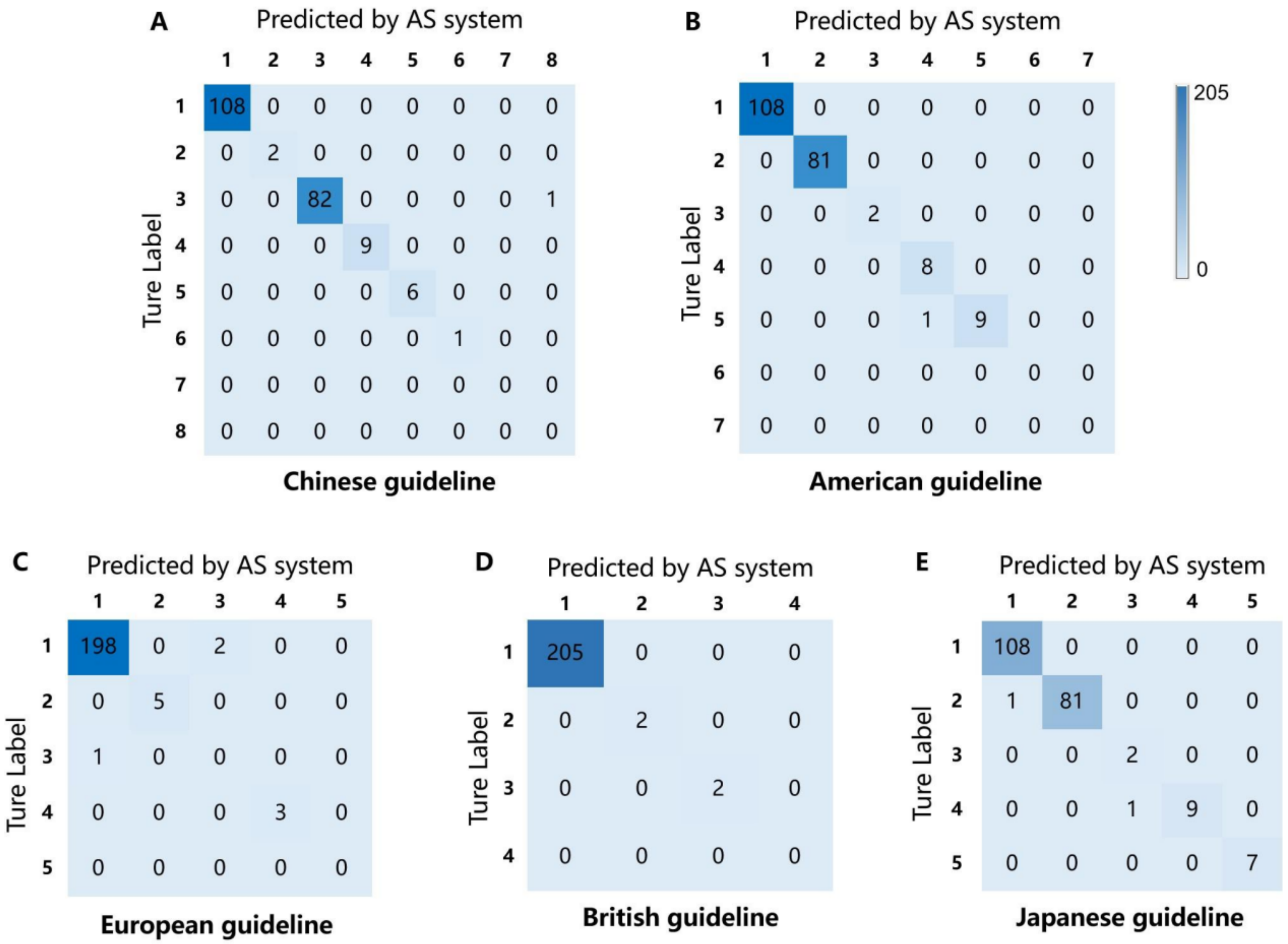

eFigure 9. The confusion matrixes illustrating detailed performance of module 2 on identifying different risk levels among patients within the external test set 2, in accordance with five distinct clinical guidelines. The labels of categories 1-8 are explained in Table S1A – S1E.

© 2023 Wu L et al. *JAMA Network Open.*

15. eFigure 10

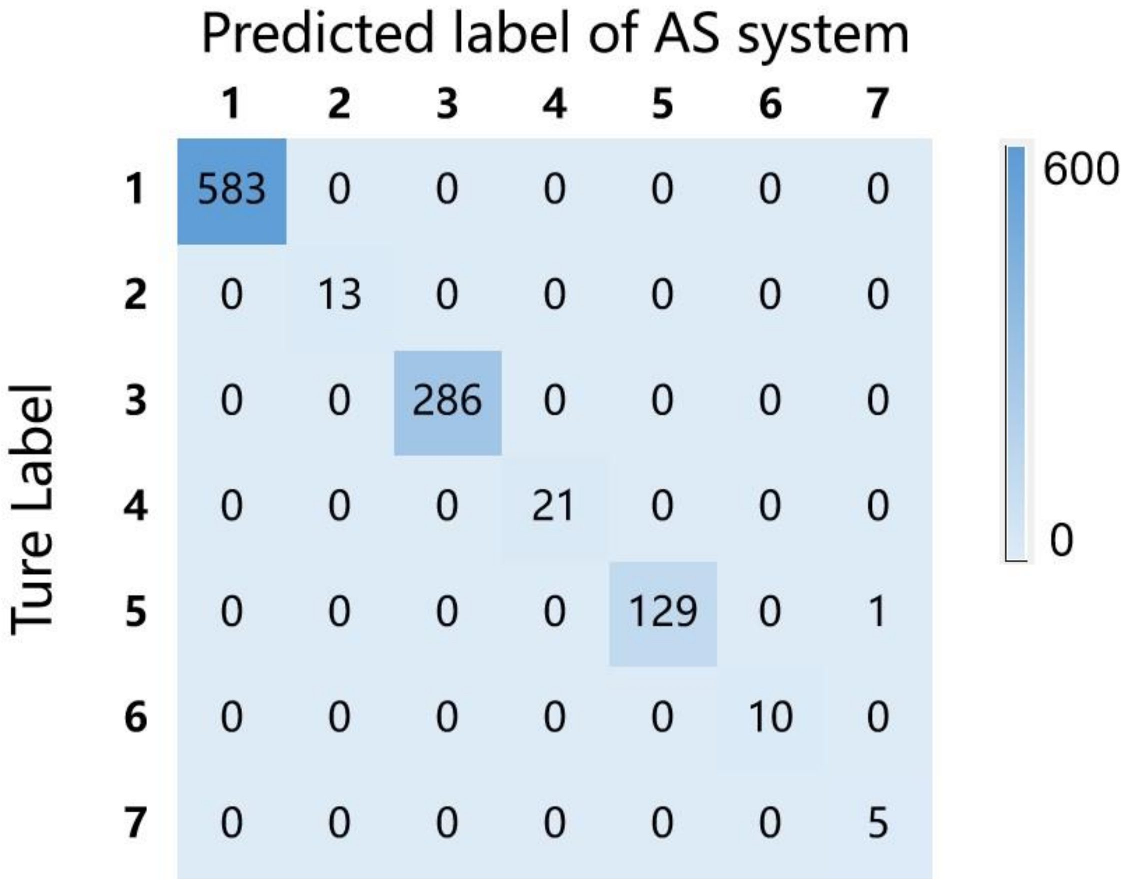

Figure S10. The confusion matrix illustrating detailed performance of automatic surveillance (AS) system on assigning surveillance interval for inpatients. The categories are based on the Chinese guideline. The labels of categories 1-7 are explained in Table S1A.

© 2023 Wu L et al. *JAMA Network Open.*

16. eFigure 11

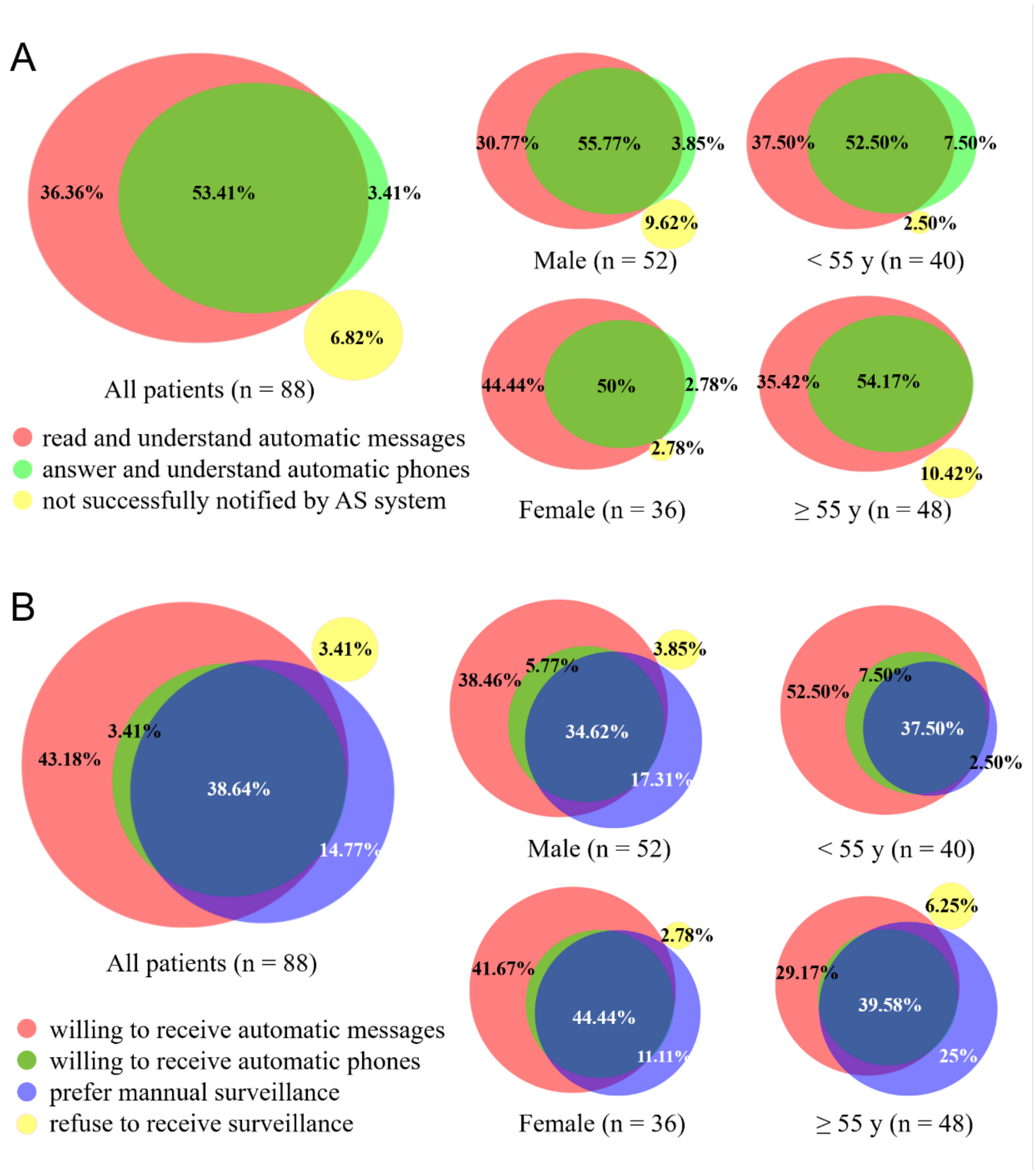

Figure S11. Details about the use of automatic surveillance (AS) system and patients' preferences in different subgroup.

© 2023 Wu L et al. *JAMA Network Open.*
