# Supplementary material for: Development and Evaluation of a Surveillance System for Follow-Up After Colorectal Polypectomy
Source: JAMA Netw Open. 2023 Sep 20;6(9):e2334822. doi: 10.1001/jamanetworkopen.2023.34822 (PMC12278838; doi:10.1001/jamanetworkopen.2023.34822)
Supplement: Supplement 2. — Data Sharing Statement [file jamanetwopen-e2334822-s002.pdf]

Data Sharing Statement

Wu. Development and Evaluation Of A Surveillance System For Follow-Up After Colorectal Polypectomy. *JAMA Netw Open*. Published online September 20, 2023. doi:10.1001/jamanetworkopen.2023.34822

Data
Data available: Yes
Data types: Deidentified participant data, Other (please specify)
Additional Information: The data and source code in this study are available in the website
How to access data: https://github.com/LeanneLL/AS-system
When available: With publication

Supporting Documents
Document types: None

Additional Information
Who can access the data: anyone requesting the data
Types of analyses: for any purpose or for a specified purpose
Mechanisms of data availability: after approval of a proposal
